# Systemic Inflammation Indices as Early Predictors of Severity in Acute Pancreatitis

**DOI:** 10.3390/jcm14155465

**Published:** 2025-08-04

**Authors:** José Francisco Araiza-Rodríguez, Brandon Bautista-Becerril, Alejandra Núñez-Venzor, Ramcés Falfán-Valencia, Asya Zubillaga-Mares, Edgar Abarca-Rojano, Samuel Sevilla-Fuentes, Luis Ángel Mendoza-Vargas, Espiridión Ramos-Martínez, Bertha Berthaúd-González, Mauricio Avila-Páez, Jennifer Manilla-González, José Manuel Guerrero Jiménez, Liceth Michelle Rodríguez Aguilar

**Affiliations:** 1 Hospital General “Dr. Manuel Gea González”, Mexico City 14080, Mexico; dr.araizar@gmail.com (J.F.A.-R.);; 2 Laboratorio de Neumogenómica, Instituto Nacional de Enfermedades Respiratorias Ismael Cosío Villegas, Mexico City 14080, Mexico; rfalfanv@iner.gob.mx; 3 Sección de Posgrado e Investigación, Escuela Superior de Medicina, Instituto Politécnico Nacional, Mexico City 11340, Mexico; 4 Hospital General de Zona 1 “Emilio Varela Luján”, Zacatecas 98000, Mexico; 5 Unidad de Medicina Experimental, Facultad de Medicina, Universidad Nacional Autónoma de México, Mexico City 04510, Mexico; 6 Hospital General de Zacatecas “Luz González Cosío”, Zacatecas 98160, Mexico; 7 Facultad de Medicina, Universidad Nacional Autónoma de México, Campus Ciudad Universitaria, Mexico City 04510, Mexico; 8 Facultad de Medicina, Universidad Popular Autónoma del Estado de Puebla, Campus Puebla, Puebla 72410, Mexico

**Keywords:** acute pancreatitis, systemic inflammatory response syndrome, inflammatory biomarkers, blood cell count, prognostic index, systemic immune-inflammation index, severity of illness index

## Abstract

**Background/Objectives**: Acute pancreatitis (AP) is a highly variable inflammatory condition that can lead to severe complications and high mortality, particularly in its severe forms. Early risk stratification is essential; however, the delayed availability of traditional scoring systems often limits its effectiveness. This study aimed to evaluate the clinical utility of systemic inflammation indices as early predictors of severity in patients with acute pancreatitis. **Methods**: A retrospective, observational study was conducted among patients diagnosed with acute pancreatitis, classified according to the revised Atlanta criteria. Upon admission, systemic inflammation indices were calculated from complete blood count parameters, including neutrophil-to-lymphocyte ratio (NLR), monocyte-to-lymphocyte ratio (MLR), systemic immune-inflammation index (SII), systemic inflammation response index (SIRI), and aggregate index of systemic inflammation (AISI). Severity was assessed using the APACHE II score. Statistical analysis involved Kruskal–Wallis tests, Dunn’s post hoc comparisons, ROC curve analysis, logistic regression for odds ratios (ORs), and Spearman correlations. **Results**: SII, NLR, MLR, SIRI, and AISI showed statistically significant associations with AP severity (*p* < 0.05). MLR and SIRI exhibited the highest predictive performance (AUC = 0.74). ORs for severe pancreatitis were: MLR = 19.10, SIRI = 7.50, NLR = 7.33, AISI = 5.12, and SII = 4.10. All four indices also demonstrated moderate positive correlations with APACHE II scores. **Conclusions**: Systemic inflammation indices are simple, cost-effective, and accessible tools that can aid in the early identification of patients at high risk for severe acute pancreatitis. Their integration into clinical practice may enhance early decision-making and improve patient outcomes.

## 1. Introduction

Acute pancreatitis (AP) is a sudden inflammatory condition of the pancreas that can range from a mild, self-limiting illness to severe, life-threatening complications. About 75% of patients experience mild episodes that resolve with supportive care, while nearly 25% develop moderate or severe forms, which are associated with a mortality rate of 15–30%, especially when persistent organ failure and pancreatic necrosis occur [1,2,3].

The updated Atlanta classification offers a reliable framework for assessing AP severity; however, accurately predicting severity upon hospital admission remains a clinical challenge. Early risk stratification is crucial, as it enables the prompt initiation of intensive care and resource allocation for high-risk patients. Traditional scoring systems, such as APACHE II, BISAP, or Ranson’s criteria, have demonstrated prognostic value; however, their use is often limited by delayed availability or complexity within the first 48 h of presentation [1,2,4].

In this context, hematological indices derived from the complete blood count (CBC)—a widely available and cost-effective laboratory test—have garnered interest as potential early biomarkers of systemic inflammation. Parameters such as the neutrophil-to-lymphocyte ratio (NLR), monocyte-to-lymphocyte ratio (MLR), systemic immune-inflammatory index (SII), systemic inflammation response index (SIRI), and aggregate index of systemic inflammation (AISI) reflect immune status and inflammatory burden, and they show promise in predicting outcomes across various inflammatory diseases [5,6,7,8,9,10,11,12,13,14].

Liu et al. (2021) [9] demonstrated that the SII is a potential indicator for predicting the severity of acute pancreatitis. Their findings suggest that SII is more sensitive and specific than NLR and PLR in predicting AP severity. Similarly, Dao et al. (2024) found that SIRI, especially when combined with the BISAP score, shows significant potential for predicting the severity of severe acute pancreatitis (SAP) in the Vietnamese clinical setting, providing valuable information for effective patient management [7,9].

However, data are still limited, and additional validation is necessary, especially in Latin-American populations, to confirm their usefulness in different clinical settings. This lack of evidence highlights the need for more validation studies in these populations to ensure wider applicability and relevance.

This study aimed to assess the clinical performance of systemic inflammation indices (SII, NLR, MLR, SIRI, AISI, RDW, RPL, RML, PCT/PCR) as early predictors of disease severity in patients with acute pancreatitis upon hospital admission. We sought to determine their association with severity classification, predictive accuracy through ROC analysis, odds ratios for severe disease, and correlation with the APACHE II score.

## 2. Materials and Methods

A retrospective, observational, analytical study was performed using simple random sampling of consecutive cases that met the inclusion criteria. The study population included patients admitted to the General and Endoscopic Surgery Department with a diagnosis of acute pancreatitis of any cause at Hospital General Dr. Manuel Gea González (Mexico City, CDMX) from 2021 to 2023.

The inclusion criteria included male and female patients aged 18 to 60 years with a diagnosis of biliary acute pancreatitis who were admitted to the previously mentioned surgical department. The exclusion criteria involved pregnant women, patients with a recent diagnosis or ongoing treatment for any type of cancer, patients who chose to discharge themselves, and cases with incomplete or inadequate clinical documentation for data extraction.

The sample size was estimated using G*Power statistical software version 3.1.9.6. An a priori calculation for a two-tailed logistic regression test was performed, utilizing a 95% confidence level and 95% statistical power. The reference proportion of 23% for complicated acute pancreatitis was derived from a study by Li et al. [15]. The final estimated sample size was 100 participants. To justify this, the effect size used was Cohen’s d = 0.5 (medium effect size), with an allocation ratio of 1:1 between groups.

Eligible participants who met the inclusion criteria were identified using the daily census of the General Surgery Department. After confirming the medical record numbers, the principal associate investigator and first co-investigator requested the relevant files from the medical archive for data collection. The following variables were extracted:

Demographic data:

- Name (later replaced by an alphanumeric code for confidentiality), sex, and age.
- Vital signs: weight, height, and body mass index (BMI).
- Laboratory results upon admission included a complete blood count and a basic metabolic panel.
- Comorbidities: diabetes mellitus, systemic arterial hypertension, and cancer.
- Hospital course: admission to the clinical ward or intensive care unit, including the date and reason for discharge (improvement or death).
- Severity assessment: APACHE II and Marshall scores, along with classification according to the revised Atlanta criteria (mild, moderately severe, severe).

For patients with multiple organ failures or complex presentations where classification might be unclear, additional clinical criteria were used. Specifically, for those with multiple organ failures, severity was determined by the duration and number of organs involved. If organ failure lasted more than 48 h, the patient was classified as severe; if it resolved within 48 h, it was considered moderately severe. In cases with borderline presentations, where it was not immediately clear whether the patient should be classified as moderately severe or severe, we examined clinical parameters such as the progression of organ dysfunction, the presence of infected pancreatic necrosis, and overall clinical deterioration. If there was significant worsening or a need for intensive care, the patient was classified as severe, even if all criteria were not fully met.

Using laboratory values collected at admission, before any treatment beyond analgesia, we ensured that blood samples were obtained upon hospital arrival, prior to administering intravenous fluids, antibiotics, or corticosteroids. This method helps reduce potential therapy-related bias and guarantees that the leukocyte subsets reflect the initial disease state.

The principal associate investigator and the first co-investigator calculated the following systemic inflammation indices.

- Neutrophil-to-lymphocyte ratio (NLR): absolute neutrophil count (ANC) divided by absolute lymphocyte count (ALC).
- Platelet-to-lymphocyte ratio (PLR): absolute platelet count (APC) divided by ALC.
- Monocyte-to-lymphocyte ratio (MLR): absolute monocyte count (AMC) divided by ALC.
- Systemic immune-inflammation index (SII): APC multiplied by NLR.
- Systemic inflammation response index (SIRI): (ANC multiplied by AMC) divided by ALC.
- Aggregate index of systemic inflammation (AISI): (ANC multiplied by AMC multiplied by APC) divided by ALC.
- Red cell distribution width (RDW): standard deviation of red blood cell volume divided by mean corpuscular volume multiplied by 100.
- Procalcitonin-to-C-reactive protein ratio (PCT/CRP index).

### Statistical Analysis

Data analysis was conducted using SPSS software, version 26 (IBM Corp., Armonk, NY, USA). Graphical representations and advanced statistical plots were generated with RStudio (version R-4.5.0) along with the following libraries: ggplot2, corrplot, pROC, Hmisc, tidyverse, rstatix, and stringi. The normality of continuous variables was tested with the Kolmogorov–Smirnov test. Due to the non-normal distribution of most variables, data are presented as medians and interquartile ranges (IQRs).

Comparisons between two groups were performed using the Mann–Whitney U test or the Fisher Exact test, while comparisons among three or more groups utilized the Kruskal–Wallis test. Post hoc pairwise comparisons were conducted using Dunn’s test with Bonferroni correction. A *p*-value of <0.05 was regarded as statistically significant in all cases. Spearman’s rank correlation coefficient was calculated to assess the relationship between plasma levels of systemic inflammation indices and various clinical and laboratory parameters.

To evaluate the discriminative ability of each inflammatory index in predicting disease severity, receiver operating characteristic (ROC) curve analysis was conducted, and the area under the curve (AUC) was determined. Optimal cut-off values were identified using the Youden index. Odds ratios (ORs) and 95% confidence intervals (CIs) were then calculated to assess the strength of the association between each index and the clinical severity categories.

## 3. Results

### 3.1. Demographic and Clinical Characteristics

Between January 2021 and December 2023, a total of 100 patient records that met the eligibility criteria were collected and analyzed. Patients were then classified according to the revised Atlanta criteria as having mild, moderately severe, or severe acute pancreatitis. The study period included both the pandemic and post-pandemic phases. To avoid potential confounding, all patients in the study tested negative for COVID-19 via PCR and also tested negative for influenza at the time of hospital admission, ensuring that the results were not affected by viral infections related to the pandemic or seasonal influenza. Additionally, none of the patients showed clinical signs or symptoms of COVID-19 or influenza, further supporting the absence of viral influence on disease severity. Of the total study population, 72% were male and 25% were female, with a median age of 43 years. Notably, the ratio of men to women among those with a severe clinical course was approximately 4:1. A detailed summary of demographic characteristics, clinical features, comorbidities, and pre-existing conditions is provided in Table 1.

To evaluate the independent predictive value of systemic inflammation indices (SII, NLR, MLR, SIRI, AISI), we conducted a multivariable logistic regression analysis, adjusting for potential confounding variables such as age, body mass index (BMI), type 2 diabetes mellitus, and systemic arterial hypertension, which showed some differences across comparison groups (Table 1).

The logistic regression model showed that after adjusting for these covariates, none of the confounding factors—age (*p* = 0.322), BMI (*p* = 0.452), type 2 diabetes mellitus (*p* = 0.674), and systemic arterial hypertension (*p* = 0.119)—were significantly associated with disease severity. This suggests that even when these variables are controlled for, the systemic inflammation indices (SII, NLR, MLR, SIRI, AISI) still significantly predict the severity of acute pancreatitis. The results highlight the robustness of these indices as independent prognostic tools, with their predictive power unaffected by factors typically linked to AP severity.

### 3.2. Systemic Inflammation Indices (RDW, SII, NLR, PLR, MLR, SIRI, AISI, PCT/CRP) and Their Association with Disease Severity in Acute Pancreatitis

To achieve our primary goal and improve the stratification of the study population, patients were divided into three groups based on disease severity, as outlined by the revised Atlanta classification: mild, moderately severe, and severe. The following results describe the effectiveness of each systemic inflammation index in relation to clinical severity.

#### 3.2.1. Systemic Immune-Inflammation Index (SII)

**Figure 1:**
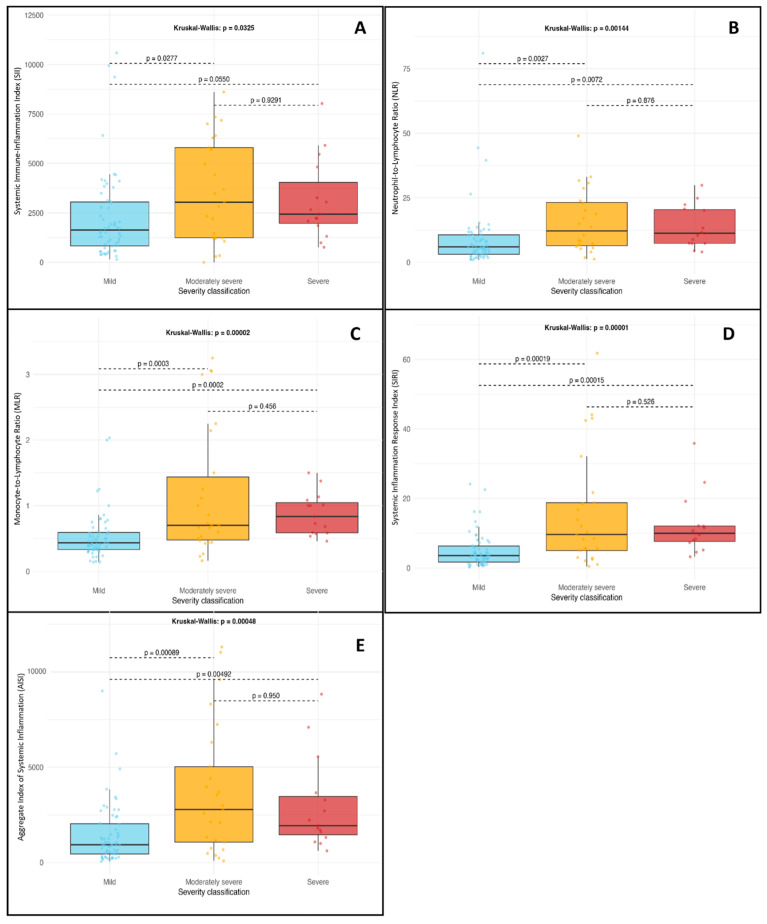
Distribution of systemic inflammation indices at admission in patients with acute pancreatitis, grouped by disease severity according to the revised Atlanta classification. (**A**) SII: Systemic Immune-Inflammation Index; (**B**) NLR: Neutrophil-to-Lymphocyte Ratio; (**C**) MLR: Monocyte-to-Lymphocyte Ratio; (**D**) SIRI: Systemic Inflammation Response Index; (**E**) AISI: Aggregate Index of Systemic Inflammation. Statistical comparisons were performed using the Kruskal–Wallis test with Dunn’s post hoc test. A *p*-value of less than 0.05 was considered statistically significant.

The Kruskal–Wallis test showed a statistically significant difference in SII values among severity groups (*p* = 0.032). Post hoc analysis using Dunn’s multiple comparisons tests found significant differences between the mild and moderately severe groups (*p* = 0.02) and between the mild and severe groups (*p* = 0.05). These results suggest that SII (Figure 1A) may be a useful early biomarker for disease severity at hospital admission.

#### 3.2.2. Neutrophil-to-Lymphocyte Ratio (NLR)

A significant association was identified between NLR values and disease severity (Kruskal–Wallis *p* = 0.001). Dunn’s test revealed notable pairwise differences between the mild and moderately severe groups (*p* = 0.002), as well as between the mild and severe groups (*p* = 0.007). These results support the role of NLR (Figure 1B) in predicting the severity of acute pancreatitis upon admission.

#### 3.2.3. Monocyte-to-Lymphocyte Ratio (MLR)

The Kruskal–Wallis analysis produced a *p*-value of <0.001, indicating a strong association between MLR and pancreatitis severity. Post hoc comparisons showed significant differences between the mild and moderately severe groups (*p* < 0.001) as well as between the mild and severe groups (*p* < 0.001), highlighting the usefulness of MLR as an early inflammatory marker (Figure 1C).

#### 3.2.4. Systemic Inflammation Response Index (SIRI)

SIRI values varied significantly among the severity groups (Kruskal–Wallis, *p* < 0.001). Dunn’s test confirmed significant differences between mild and moderately severe (*p* < 0.001) as well as mild and severe (*p* < 0.001) conditions. These results indicate that SIRI (Figure 1D) may effectively distinguish patients at higher risk of clinical deterioration.

#### 3.2.5. Aggregate Index of Systemic Inflammation (AISI)

AISI also showed significant variation across severity categories (Kruskal–Wallis *p* < 0.001). Post hoc comparisons revealed significant differences between the mild and moderately severe groups (*p* < 0.001) and between the mild and severe groups (*p* = 0.004). This composite index (Figure 1E) appears promising for assessing the systemic inflammatory burden in patients with acute pancreatitis.

#### 3.2.6. Procalcitonin-to-C-Reactive Protein Ratio (PCT/CRP)

No significant association was found between PCT/CRP and disease severity (Kruskal–Wallis *p* = 0.795). Dunn’s post hoc test showed no significant pairwise differences. The lack of statistical significance may be due to the small number of patients with available biomarker data (*n* = 17). Due to its exploratory nature and limited statistical power, this analysis is included in Supplementary Figure S1A.

#### 3.2.7. Platelet-to-Lymphocyte Ratio (PLR)

PLR did not show significant differences among severity groups (Kruskal–Wallis *p* = 0.348). Pairwise comparisons also were not significant: mild vs. moderately severe (*p* = 0.086), mild vs. severe (*p* = 0.150), and moderately severe vs. severe (*p* = 0.864). Since no clear association was demonstrated, PLR data are presented in Supplementary Figure S1B.

#### 3.2.8. Red Cell Distribution Width (RDW)

Similarly, RDW values did not show a significant association with clinical severity (Kruskal–Wallis *p* = 0.588). Post hoc tests revealed no significant differences among the groups. This analysis is illustrated in Supplementary Figure S1C.

### 3.3. Systemic Inflammation Indices (SII, NLR, MLR, SIRI, and AISI) as Predictors of Severity in Acute Pancreatitis

To achieve our primary goal, we assessed the predictive ability of systemic inflammation indices that have demonstrated a statistically significant link to disease severity in previous analyses: SII, NLR, MLR, SIRI, and AISI. Initially, indices like RDW, PCT/CRP, and PLR were included in the study because of their link to inflammation and potential use in assessing disease severity. However, since no significant differences appeared in our main analyses, we decided to focus on the five key indices (SII, NLR, MLR, SIRI, and AISI) that showed stronger links with disease severity. Their ability to distinguish between mild to moderate and severe acute pancreatitis at the time of hospital admission was evaluated using ROC curve analysis. The best cut-off values were identified using the Youden index.

#### 3.3.1. Monocyte-to-Lymphocyte Ratio (MLR)

**Figure 2:**
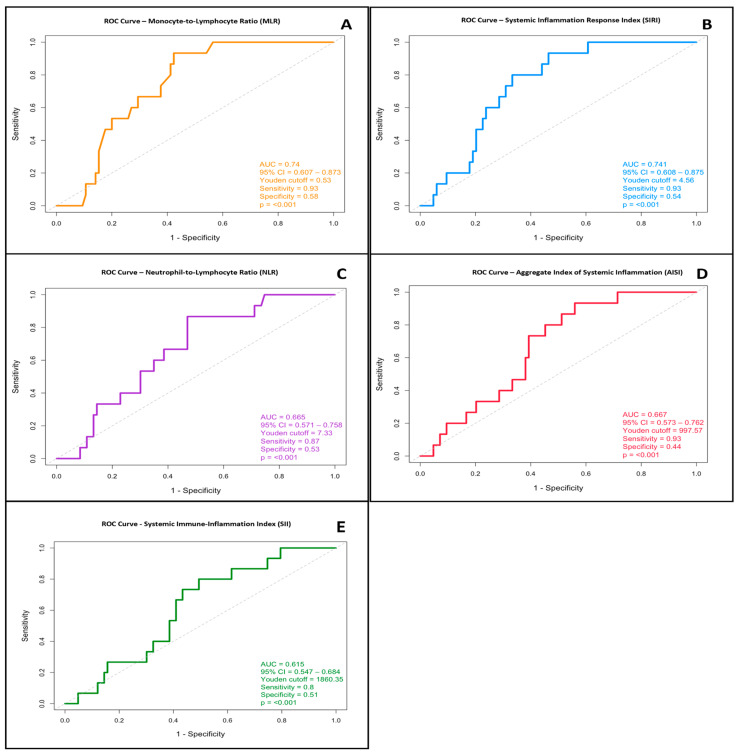
Receiver operating characteristic (ROC) curves for systemic inflammation indices that have a significant association with acute pancreatitis severity at admission. (**A**) MLR: Monocyte-to-Lymphocyte Ratio (AUC = 0.740, 95% CI: 0.607–0.873, *p* < 0.001; optimal cut-off: 0.53). (**B**) SIRI: Systemic Inflammation Response Index (AUC = 0.741, 95% CI: 0.608–0.875, *p* < 0.001; cut-off: 4.56). (**C**) NLR: Neutrophil-to-Lymphocyte Ratio (AUC = 0.665, 95% CI: 0.571–0.758, *p* < 0.001; cut-off: 7.33). (**D**) AISI: Aggregate Index of Systemic Inflammation (AUC = 0.667, 95% CI: 0.573–0.762, *p* < 0.001; cut-off: 997.57). (**E**) SII: Systemic Immune-Inflammation Index (AUC = 0.615, 95% CI: 0.547–0.684, *p* ≤ 0.001; cut-off: 1860.35).

The MLR index showed strong predictive ability, with an odds ratio (OR) of 19.10 at a Youden-derived cutoff of 0.53. The AUC for MLR was 0.740 (95% CI: 0.607–0.873; *p* < 0.001), indicating moderate discriminative power. The index demonstrated high sensitivity and specificity for detecting severe cases of acute pancreatitis, as confirmed by the statistical analysis. The optimal cutoff point identified using the Youden index was 0.58, supporting its usefulness as an early predictor of clinical severity (Figure 2A).

#### 3.3.2. Systemic Inflammation Response Index (SIRI)

SIRI showed comparable performance, with an AUC of 0.741 (95% CI: 0.608–0.875; *p* = 0.001), indicating reliable discrimination between patients with severe and non-severe disease. The most informative threshold was 4.56, suggesting that elevated SIRI values at admission may act as an early warning sign (Figure 2B).

#### 3.3.3. Neutrophil-to-Lymphocyte Ratio (NLR)

Although the AUC for NLR was 0.665, its confidence interval (95% CI: 0.571–0.758) and statistical significance (*p* < 0.001) support its importance in clinical assessment. The optimal cutoff value was determined to be 7.33. Despite its moderate ability to discriminate, NLR remains a simple and accessible index for early risk stratification (Figure 2C).

#### 3.3.4. Aggregate Index of Systemic Inflammation (AISI)

AISI achieved an AUC of 0.667 (95% CI: 0.573–0.762; *p* < 0.001). Although the discriminative ability was moderate, its statistical significance and acceptable confidence interval emphasize its potential in identifying patients at higher risk. The optimal cut-off point for AISI was 997.57 (Figure 2D).

#### 3.3.5. Systemic Immune-Inflammation Index (SII)

ROC analysis produced an AUC of 0.615 (95% CI: 0.548–0.684; *p* = 0.0009), with an optimal cutoff value of 1860. This suggests a statistically significant, though moderate, ability of SII to distinguish severe cases. The analysis is shown in Figure 2E.

### 3.4. Systemic Inflammation Indices (NLR, MLR, SIRI, and AISI) and Odds Ratios for Predicting Severity in Acute Pancreatitis

To further support the predictive value of systemic inflammation indices, we calculated the odds ratios (ORs) for markers that showed a significant association with disease severity and acceptable discriminative ability. The monocyte-to-lymphocyte ratio (MLR) demonstrated the highest predictive strength, with an odds ratio (OR) of 19.10 using a Youden-derived cut-off of 0.53. This was followed by the systemic inflammation response index (SIRI), with an OR of 7.50 at a cut-off of 4.56, and the neutrophil-to-lymphocyte ratio (NLR), with an OR of 7.33 at a cut-off of 7.33. The aggregate index of systemic inflammation (AISI) and the systemic immune-inflammation index (SII) also showed significant predictive capacity, with ORs of 5.12 and 4.10 at cut-offs of 997.57 and 1860.35, respectively.

These results emphasize the clinical usefulness of these indices as early and easily accessible tools for identifying patients at high risk of developing severe acute pancreatitis. Complete details are provided in Table 2.

### 3.5. Systemic Inflammation Indices (RNL, MLR, SIRI, and AISI) and Odds Ratios for Predicting Severity in Acute Pancreatitis

**Figure 3:**
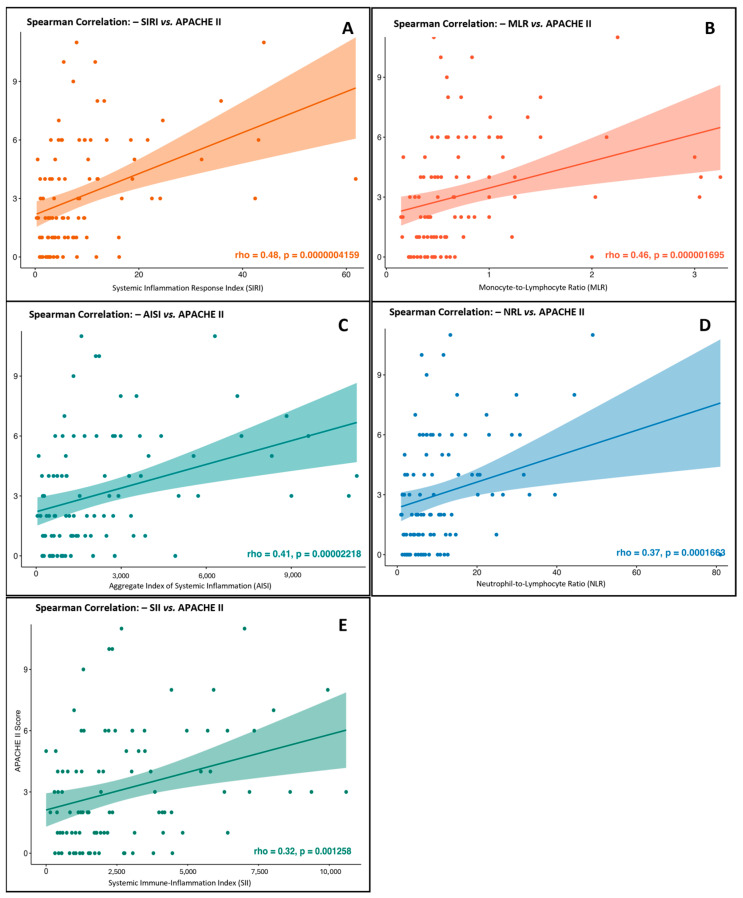
Correlation between systemic inflammation indices and APACHE II severity scores in patients with acute pancreatitis. (**A**) SIRI: Systemic Inflammation Response Index (Spearman’s Rho = 0.483, *p* = 0.001). (**B**) MLR: Monocyte-to-Lymphocyte Ratio (Spearman’s Rho = 0.460, *p* = 0.001). (**C**) AISI: Aggregate Index of Systemic Inflammation (Spearman’s Rho = 0.413, *p* = 0.001). (**D**) NLR: Neutrophil-to-Lymphocyte Ratio (Spearman’s Rho = 0.379, *p* = 0.001). (**E**) SII: Systemic Immune-Inflammation Index (Spearman’s Rho = 0.320, *p* = 0.0013).

We assessed the correlation between systemic inflammation indices, which showed significant associations with pancreatitis severity, and the APACHE II clinical severity score to evaluate their agreement with established prognostic scales. Using Spearman’s non-parametric test, we found a statistically significant, moderate, positive correlation between the neutrophil-to-lymphocyte ratio (NLR) and the APACHE score (Rho = 0.379, *p* < 0.001), indicating that higher NLR values were associated with greater severity (Figure 3A). A stronger correlation was observed for the monocyte-to-lymphocyte ratio (MLR), with a Rho of 0.460 (*p* < 0.001), supporting its clinical relevance with APACHE-based severity assessments (Figure 3B). The systemic inflammation response index (SIRI) showed the highest correlation in this analysis (Rho = 0.483, *p* < 0.001), reinforcing its role as a strong marker of systemic burden and predictive severity (Figure 3C). Similarly, the aggregate index of systemic inflammation (AISI) demonstrated a significant positive correlation (Rho = 0.413, *p* = 0.001), further supporting its clinical usefulness in reflecting disease progression and severity (Figure 3D). Lastly, the systemic immune-inflammation index (SII) also showed a significant, though slightly weaker, correlation with APACHE II (Rho = 0.320, *p* = 0.0013), suggesting its potential value in prognostic evaluation (Figure 3E). These results highlight the consistency between systemic inflammation indices and validated clinical severity tools in acute pancreatitis, with the identified cut-offs providing important thresholds for early risk assessment and triage decisions to support timely clinical interventions.

## 4. Discussion

Our study evaluated the usefulness of different systemic inflammation indices as early indicators of severity in patients with acute pancreatitis. The findings indicated that indices such as the Systemic Immune-Inflammatory Index (SII), Neutrophil-to-Lymphocyte Ratio (NLR), Monocyte-to-Lymphocyte Ratio (M/LR), Systemic Inflammatory Response Index (SIRI), and Aggregate Inflammation Score (AISI) were significantly associated with disease severity (*p* < 0.05). Notably, the MLR and SIRI indices showed moderate ability to distinguish patients at high risk for severe disease, with areas under the curve (AUC) of 0.740 and 0.741, respectively. These AUC values suggest they are effective as reliable markers for early risk stratification in acute pancreatitis.

These findings are consistent with those of Zhang et al. [12], who identified SIRI as a relevant marker for predicting AP severity, with an AUC of 0.785, slightly higher than the value observed in our study (AUC = 0.741). Additionally, the SIRI, NLR, and SII indices showed significant associations with disease severity in both our study and Zhang et al.’s research. These findings highlight the clinical usefulness of these indices as valuable tools for early stratification of AP severity, enabling the timely identification of patients who may need intensive care or more aggressive treatment. Incorporating them into clinical practice could significantly improve decision-making, allowing for earlier detection of high-risk patients who could benefit from more targeted management, including ICU admission.

Liu et al. [9] demonstrated that an SII ≥ 2207.53 is significantly associated with severe AP, with a sensitivity of 92.9%, specificity of 87.7%, and an AUC of 0.920. Their findings indicate that SII is more predictive of AP severity than other indices like PLR and NLR. Similarly, Dao et al. [7] found that the SIRI index was significantly higher in patients with severe AP (median SIRI = 12.0) compared to those with mild cases (*p* < 0.001). SIRI was identified as an independent predictor of severe AP (OR = 1.623), and when combined with the BISAP score, it improved predictive ability, achieving a sensitivity of 90.91%.

In a recent study by Li et al. (2021), four inflammation-based models were evaluated for predicting severe acute pancreatitis (SAP). These models, which incorporated different combinations of liver fat, PCT, NLR, SII, and other inflammatory markers, showed AUROCs ranging from 0.771 to 0.795. Specifically, Model 2, which included SII, reported an AUC of 0.780 (95% CI: 0.708–0.852). In comparison, our study demonstrated AUROCs for SII, SIRI, and MLR of 0.62, 0.67, and 0.74, respectively, indicating that while our results are consistent with those found in other cohorts [15].

These findings further support the potential of systemic inflammation indices such as SII, SIRI, NLR, MLR, and AISI in early AP severity assessment, offering valuable tools for clinical decision-making. However, it is important to note that these indices are most effective when used alongside established scoring systems like APACHE II or BISAP, rather than replacing them. While they provide useful insights into disease severity, their usefulness is maximized when combined with more comprehensive scoring systems, which enhance overall predictive accuracy and assist in clinical management.

By contrast, other indices such as Red Cell Distribution Width (RDW), Platelet-to-Lymphocyte Ratio (RPL), and Procalcitonin-to-C-Reactive Protein Ratio (PCT/PCR) did not demonstrate a statistically significant link with AP severity. Notably, the PCT/PCR index showed limitations, likely due to the small number of patients measured for these markers, which may have compromised its statistical power. Future prospective studies with larger cohorts and more rigorous designs could help clarify the potential role of these markers in AP management.

The monocyte-to-lymphocyte ratio (MLR) showed the strongest predictive power, with an odds ratio (OR) of 19.10 at a Youden-derived cut-off of 0.53. Although the 95% confidence interval (CI: 3.58–353.0) is broad, this does not lessen MLR’s potential as a reliable predictor. The wide confidence interval likely results from the small sample size and data variability, which are common in studies with limited participants. This highlights the importance of future research with larger cohorts to better refine these estimates and more accurately confirm MLR’s predictive ability.

Furthermore, the indices NLR, RML, SIRI, and AISI demonstrated strong correlations with the APACHE II score, a well-validated method for evaluating AP severity. The most prominent correlation was seen with the SIRI index (Rho = 0.483, *p* = 0.001), indicating that this marker could be added to existing prognostic scales to improve their predictive accuracy. This is especially important because, although the APACHE II score is accurate, it requires the assessment of many physiological and biochemical parameters, which can delay prompt intervention, particularly in resource-limited settings.

Systemic inflammation indices such as SII, SIRI, and MLR, derived from routine blood tests, provide a more accessible and cost-effective option for early risk assessment. These indices can be integrated into clinical workflows alongside APACHE II or BISAP scores to support clinicians in early decision-making. For example, clinicians could use systemic inflammation indices as an initial screening tool to quickly identify patients at high risk of severe disease before the complete APACHE II or BISAP score is available. Once the severity is more precisely determined, these indices can help further refine management strategies, enabling prompt interventions in high-risk patients, especially in settings with limited access to advanced imaging and resources. Therefore, incorporating these indices could improve the accuracy and speed of early diagnosis and enhance overall management of AP patients.

These findings align with the research by Altuğ Ertuğrul et al. [11] Biyik et al. [10], and Liu et al. [9], who have demonstrated the usefulness of systemic inflammation indices in predicting severity in other inflammatory conditions, including sepsis, cardiovascular diseases, and cancer. The activation of systemic inflammatory responses plays a crucial role in the progression of AP, and these indices may reflect the extent of immune dysfunction and tissue damage. Consequently, their use in AP could offer a valuable alternative for enhancing clinical decision-making and optimizing hospital resource allocation, especially in settings where access to advanced imaging or specific biomarkers is limited.

Our study makes a significant contribution to the current literature by refining how systemic inflammation indices are used to predict the severity of acute pancreatitis (AP). While previous research has shown the potential of indices such as SIRI, SII, and NLR, our work advances these findings in several important ways. First, we offer more accurate cut-off values for SIRI and RML, improving their effectiveness in identifying patients at high risk of severe disease. In our study, the identified MLR cut-off demonstrated notable predictive value for the severity of acute pancreatitis. Compared to thresholds reported in earlier studies, such as those by Zhang et al. (2021) and Liu et al. (2021) [9,12], our results are consistent with the trend of using systemic inflammation indices to categorize disease severity. Zhang et al. (2021) found that high SII values (≥113.4) were linked to increased mortality in ICU patients with AP, with a hazard ratio (HR) of 2.78 (95% CI: 1.49–5.19). Similarly, Liu et al. (2021) reported that a SII cut-off of ≥2207.53 had high sensitivity (92.9%) for predicting severe acute pancreatitis, with an AUC of 0.920 [9,12]. Our MLR cut-off, although different from these studies, adds to the growing body of evidence supporting systemic inflammation indices as reliable predictors of severe disease in AP. These comparisons highlight the potential clinical importance and consistency of MLR as an addition to established severity scores.

This improved precision provides better prognostic accuracy, which is essential for early risk stratification in clinical practice. Second, including a diverse patient population across multiple hospitals in Mexico enhances the generalizability of our findings, demonstrating the applicability of these indices in different healthcare environments. Lastly, our study’s combination of systemic inflammation indices with the APACHE II score introduces a new approach by merging simple, cost-effective biomarkers with an established severity classification system, further enhancing the prediction of AP severity and supporting prompt clinical decision-making.

Nonetheless, our study shows that SII, RNL, RML, SIRI, and AISI indices are useful markers for predicting AP severity, especially RML and SIRI because of their high sensitivity and moderate correlation with the APACHE II score. This study has limitations. As a single-center study conducted in Mexico, the applicability of our findings may be affected by cultural, genetic, and healthcare system differences. Although the results provide valuable insights into using systemic inflammation indices for early risk assessment in acute pancreatitis, more prospective and multicenter studies are necessary to confirm these findings and evaluate their relevance in different clinical settings, which could eventually lead to their inclusion in AP management guidelines.

Additionally, the small number of patients with available PCR and PCT measurements limited their inclusion in the primary analysis. Since these biomarkers did not show significant associations with disease severity, they were excluded from the main analyses and included in the Supplementary Materials. Future studies with larger, more comprehensive datasets are necessary to better assess the clinical relevance of PCR and PCT in acute pancreatitis.

Finally, future studies could explore combining systemic inflammation indices with biochemical markers like BUN and creatinine or with validated severity scores such as BISAP to improve predictive accuracy and clinical usefulness. We selected the Atlanta classification as the main severity scoring system because it is the most commonly used in our hospital. We plan to incorporate BISAP and other markers in future research to improve clinical decision-making tools. This study has emphasized key findings, but there are still significant areas for further investigation. Future research should aim to validate these indices in larger, multicenter cohorts and examine their integration with other severity scores and biochemical markers. Long-term studies are also necessary to evaluate the practical utility of these indices in guiding clinical decisions.

## 5. Conclusions

Systemic inflammation indices are affordable, easily accessible tools that show promising potential for predicting disease severity in acute pancreatitis early on. In our study, SII, NLR, SIRI, RML, and AISI showed significant associations with clinical severity. Of these, RML and SIRI had the highest predictive accuracy (AUC = 0.74). Using optimal thresholds, higher values of RML, SIRI, NLR, and AISI were associated with significantly greater odds of severe acute pancreatitis, with odds ratios (ORs) of 19.10, 7.50, 7.33, and 5.12, respectively. Additionally, all four indices had moderate, statistically significant correlations with APACHE II scores. These results support the usefulness of systemic inflammation indices as quick, supplementary markers for early risk assessment and decision-making in patients with acute pancreatitis.

## Figures and Tables

**Table 1:** Demographic, anthropometric, and comorbidity characteristics by severity group based on the revised Atlanta classification.

Characteristic	Mild (*n* = 59)	Moderately Severe (*n* = 26)	Severe (*n* = 15)	*p*-Value
Age (years)	36 (26–43)	48 (33–52)	45 (35–52)	0.011
Sex				
Male	44 (74.6%)	17 (65.4%)	7 (46.7%)	0.111
Female	15 (25.4%)	9 (34.6%)	8 (53.3%)	
Body Mass Index (kg/m^2^)	28.84 (25.71–31.25)	29.14 (25.09–32.34)	30.91 (27.94–34.04)	0.899
Type 2 Diabetes Mellitus	13 (22.0%)	9 (34.6%)	8 (53.3%)	0.052
Systemic Arterial Hypertension	12 (20.3%)	9 (34.6%)	6 (40.0%)	0.018

Data expressed as median (interquartile range) or absolute number (percentage). *p*-values were calculated using the Kruskal–Wallis or Fisher Exact test.

**Table 2:** Odds ratios for severe acute pancreatitis based on systemic inflammation indices using Youden-derived cutoff values.

Index	Youden Cutoff	Odds Ratio	95% CI	*p*-Value
AISI	997.57	5.12	1.31–34.00	0.038
RNL	7.33	7.33	1.87–48.8	0.011
SIRI	4.56	7.50	1.92–49.90	0.018
SII	1860.35	4.10	1.20–18.90	0.038
MLR	0.53	19.10	3.58–353.00	0.005

Odds ratios (OR) and 95% confidence intervals (CI) were calculated using binary logistic regression based on severity classification (severe vs. non-severe) according to the revised Atlanta criteria. Cut-off values were determined using the Youden index from ROC curve analysis. All indices were recorded upon hospital admission. AISI: Aggregate Inflammation Score. RNL: Ratio of Neutrophils to Lymphocytes. SIRI: Systemic Inflammatory Response Index. SII: Systemic Immune-Inflammatory Index. MLR: Monocyte-to-Lymphocyte Ratio.

## Data Availability

The original contributions presented in this study are included in the article/Supplementary Materials. Further inquiries can be directed to the corresponding author.

## Supplementary Materials

The following supporting information can be downloaded at: https://www.mdpi.com/article/10.3390/jcm14155465/s1, Figure S1: Distribution of selected inflammation-related biomarkers at admission in patients with acute pancreatitis, stratified by disease severity according to the revised Atlanta classification. (A) Procalcitonin-to-C-reactive Protein Ratio (PCT/CRP); (B) Platelet-to-Lymphocyte Ratio (PLR); (C) Red Cell Distribution Width (RDW). No significant association was observed between these biomarkers and disease severity.
