# Supplementary material for: Systemic Inflammation Indices as Early Predictors of Severity in Acute Pancreatitis
Source: J Clin Med. 2025 Aug 4;14(15):5465. doi: 10.3390/jcm14155465 (PMC12347433; doi:10.3390/jcm14155465)
Supplement: Supplementary file 1 [file jcm-14-05465-s001.zip › jcm-3735562-supplementary.pdf]

Supplementary Figures

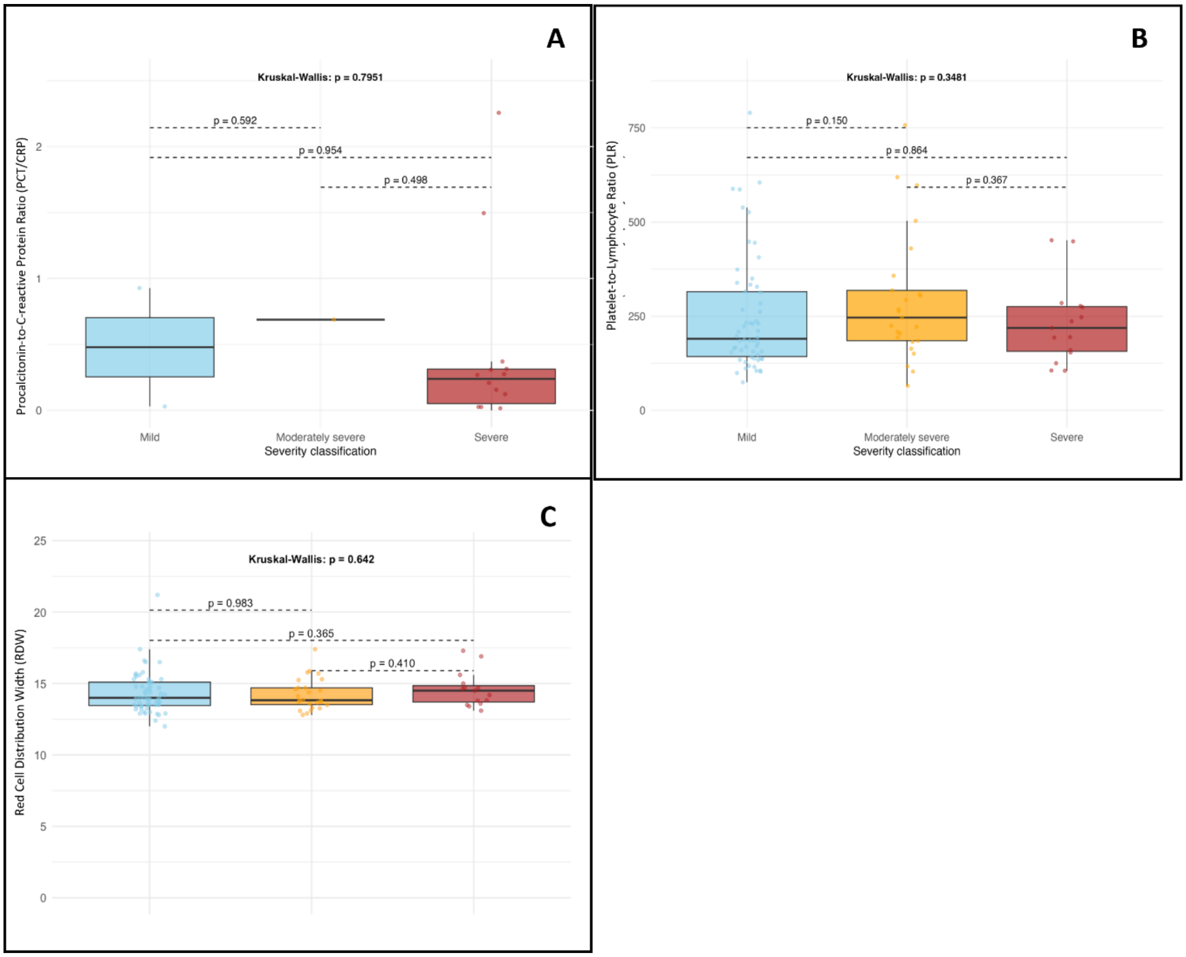

.

Figure S1. *Distribution of selected inflammation-related biomarkers at admission in patients with acute pancreatitis, stratified by disease severity according to the revised Atlanta classification. (A) Procalcitonin-to-C-reactive Protein Ratio (PCT/CRP); (B) Platelet-to-Lymphocyte Ratio (PLR); (C) Red Cell Distribution Width (RDW). No significant association was observed between these biomarkers and disease severity.*
